# Items

**Published:** 1906-01

**Authors:** 


### ITEMS.

---

MESSRS. Battle & Co., Saint Louis, have recently issued the eighth of their series of twelve illustrations of intestinal parasites, which will be sent to any physician on application. Address the publishers, 2001 Locust Street, Saint Louis, Mo.

---

APOLLINARIS continues to be the favorite table water, in spite of all rivals, and they are not few in number. It is the most palatable of all the bottled waters, its own natural carbonic acid gas giving it just enough sparkle to suit the average taste. As a drink in the sick room it is unrivalled, a fact that every physician should remember.

---

THE Antikamnia Chemical Company of Saint Louis, has sent out an attractive calendar for 1906. The obverse is a reproduction of a water color by Dietrich of two little sisters of charity each holding an antikamnia tablet, and is entitled "Opposed to Pain" The reverse, besides the calendar, is made up of a miscellaneous group of material relating to the use of antikamnia. Duplicates will be sent by mail on receipt by the company of ten cents to cover postage.

---

MESSRS. William R. Warner & Co., Philadelphia, sent out as a holiday souvenir, a reproduction on crystalloid of the celebrated painting, "Beatrice" by Weiss. The original is in the art collection of Mr. Whitehead, of Newark, N. J.

Fourteen separate printings were involved in this reproduction, and it is one of the handsomest little plaques we have seen in many a day. Its artistic merit cannot but be apreciated by everyone who receives this delicate "compliment of the season."

